# Characterization of a community-acquired-MRSA USA300 isolate from a river sample in Austria and whole genome sequence based comparison to a diverse collection of USA300 isolates

**DOI:** 10.1038/s41598-018-27781-8

**Published:** 2018-06-21

**Authors:** Sarah Lepuschitz, Steliana Huhulescu, Patrick Hyden, Burkhard Springer, Thomas Rattei, Franz Allerberger, Robert L. Mach, Werner Ruppitsch

**Affiliations:** 1 Austrian Agency for Health and Food Safety, National Reference Laboratory for Coagulase Positive Staphylococci including Staphylococcus aureus, Graz, Austria; 2 Vienna University of Technology, Research Area of Biochemical Technology, Institute of Chemical, Biological and Environmental Engineering, Vienna, Austria; 3 University of Vienna, Department of Microbiology and Ecosystem Science, Vienna, Austria; 4 University of Natural Resources and Life Sciences, Department of Biotechnology, Vienna, Austria

## Abstract

The increasing emergence of multi-resistant bacteria in healthcare settings, in the community and in the environment represents a major health threat worldwide. In 2016, we started a pilot project to investigate antimicrobial resistance in surface water. Bacteria were enriched, cultivated on selective chromogenic media and species identification was carried out by MALDI-TOF analysis. From a river in southern Austria a methicillin resistant *Staphylococcus aureus* (MRSA) was isolated. Whole genome sequence analysis identified the isolate as ST8, *spa* type t008, SCCmecIV, PVL and ACME positive, which are main features of CA-MRSA USA300. Whole genome based cgMLST of the water isolate and comparison to 18 clinical MRSA USA300 isolates from the Austrian national reference laboratory for coagulase positive staphylococci originating from 2004, 2005 and 2016 and sequences of 146 USA300 isolates arbitrarily retrieved from the Sequence Read Archive revealed a close relatedness to a clinical isolate from Austria. The presence of a CA-MRSA USA300 isolate in an aquatic environment might pose a public health risk by serving as a potential source of infection or a source for emergence of new pathogenic MRSA clones.

## Introduction

Methicillin resistant strains of *Staphylococcus aureus* (MRSA) are the leading cause of nosocomial infections^1^. Manifestations vary from minor skin infection to fatal disease. MRSA have an outstanding ability to acquire antibiotic resistance genes leading to resistance to multiple antibiotic classes^2^. Since the late 1960s hospital acquired MRSA (HA-MRSA) became endemic in hospitals worldwide^3^. In the 1990s new clones affecting also healthy individuals in the community, featuring increased virulence as well as the power to spread easily, arose^4^. This so called community acquired MRSA (CA-MRSA) became prevalent worldwide with predominant clones in certain geographic areas. Clonal Complex (CC) 1 (USA400) and CC8 (USA300) are the major lineages in the United States, CC80 is the predominant lineage in Europe, CC59 and CC30 appear in Asia and the southwest Pacific^5,6^. CA-MRSA is now epidemic in the United States mainly due to dissemination of the USA300 clone which belongs to multi locus sequence type (MLST) 8/SCCmec IV and harbours the *luk*S-*luk*F genes, encoding the Panton-Valentine leukocidin (PVL) and the arginine catabolic mobile element (ACME) cluster^7^. While virulence of these strains enhances dissemination, the potential to acquire resistance to multiple antibiotic classes hinders treatment of MRSA infections^1^. CA-MRSA clones are not restricted to a geographical region. The ability of geographically predominant CA-MRSA clones to spread worldwide and to cause infections on other continents has been demonstrated^2^. In several European countries, where ST80 still represents the prevalent CA-MRSA clone, also infections due to the North American USA300 clone have been reported^8–11^. Consequently the identification of new potential infection sources is essential for infection control.

Antimicrobial resistance (AMR) is an increasing global problem and the fact that resistant pathogens are not restricted to clinical settings but can be increasingly found in the environment is alarming^12–14^. In aquatic systems, horizontal gene transfer (HGT) of resistance genes between bacteria leads to the evolution of AMR bacteria, which in turn find back into clinical settings. AMR dissemination of resistant strains occur through direct contact of humans to the AMR bacteria in the aquatic system or from contact to resistant environmental bacteria which pass resistance genes to human or animal pathogens^15^. So called high risk clones (HRCs), which are characterized by enhanced virulence and multiple antibiotic resistances, pose a serious public health risk^16^. Aanensen *et al*.^8^ identified three key elements to tackle the public health threat caused by HRCs: genetic population structure and identification of HRCs, assessment of risks posed by virulence and resistance determinants and risk management by implementation of prevention and control strategies.

The Austrian Agency for Health and Food Safety, the major Austrian organization responsible for consumer protection and public health, started a pilot project analysing surface water samples from rivers and bathing sites with the aim to identify possible public health risks due to AMR HRCs in 2016^17^. Environmental screening for HRCs and high discriminatory typing identified a CA-USA300 isolate in a river water sample in Austria in 2016.

In this report we describe a detailed characterization of this USA300 MRSA isolate and its phylogenetic relatedness to clinical CA-MRSA USA300 isolates.

## Results

Screening of the 12 water samples for MRSA revealed one sample from a river in southern Austria (46°38′14″N, 14°17′44″E) positive for MRSA (sample ID: W1). Whole genome sequencing (WGS) identified the river water isolate W1 as a USA300 strain. The isolate showed classical multi locus sequence type (MLST) sequence type (ST) 8, *spa* type t008, harboured SCCmecIV, the Panton-Valentine leucocidin (PVL) genes, the arginine catabolic mobile element (ACME) cluster and USA300 specific *cap5* mutations.

### Genetic comparison of ST8 isolates

To assess relationship of the river water isolate W1 to clinical and food associated isolates, 18 clinical isolates from the Austrian national reference laboratory for coagulase positive staphylococci were characterized by WGS and sequences of 146 arbitrarily chosen USA300 isolates were retrieved from the Sequence Read Archive (SRA).

From the comparison of isolate W1 to in total 164 isolates, isolate W1 was indistinguishable from 99 USA300 isolates concerning ST8, *spa* type t008, SCCmecIV, PVL genes, the ACME cluster and *cap5* mutations. Eighteen isolates carried no ACME cluster, six isolates carried no PVL and four isolates carried no ACME cluster and no PVL. Thirty isolates had ST8, carried the ACME cluster and PVL but had different *spa* types: t024 (n = 5), t063 (n = 2), t068 (n = 1), t121 (n = 6), t16000 (n = 1), t2031 (n = 11), t622 (n = 3), t681 (n = 1). One isolate had ST8, *spa* type t622, PVL and no ACME cluster; one isolate had ST8, *spa* type t203, the ACME cluster and no PVL; four isolates had ST8, different *spa* types (t024 (n = 1), t190 (n = 2), t5271 (n = 1)) and carried no ACME cluster and no PVL. One isolate had ST3885, *spa* type t008, the PVL genes and the ACME cluster.

**Figure 1:**
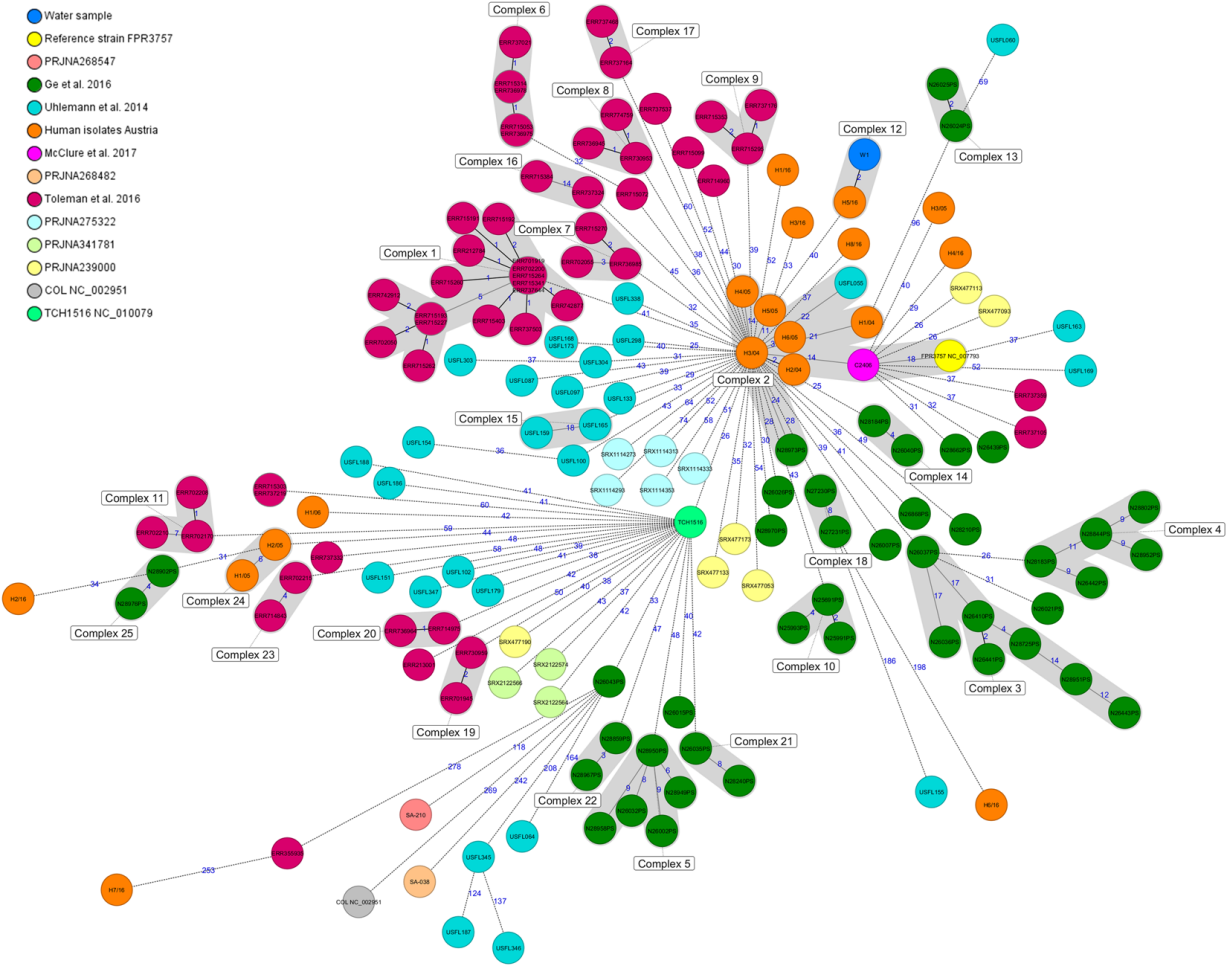
Minimum spanning tree for 165 MRSA isolates based on the cgMLST of *S. aureus*. Colours correspond to the origin of the samples. Each circle represents isolates with an allelic profile based on the sequence of 1,861 core genome targets. Blue numbers refer to the allelic differences between two isolates. Isolates with closely related genotypes were identified with a maximum of 24 allelic differences and are shaded in grey.

**Figure 2:**
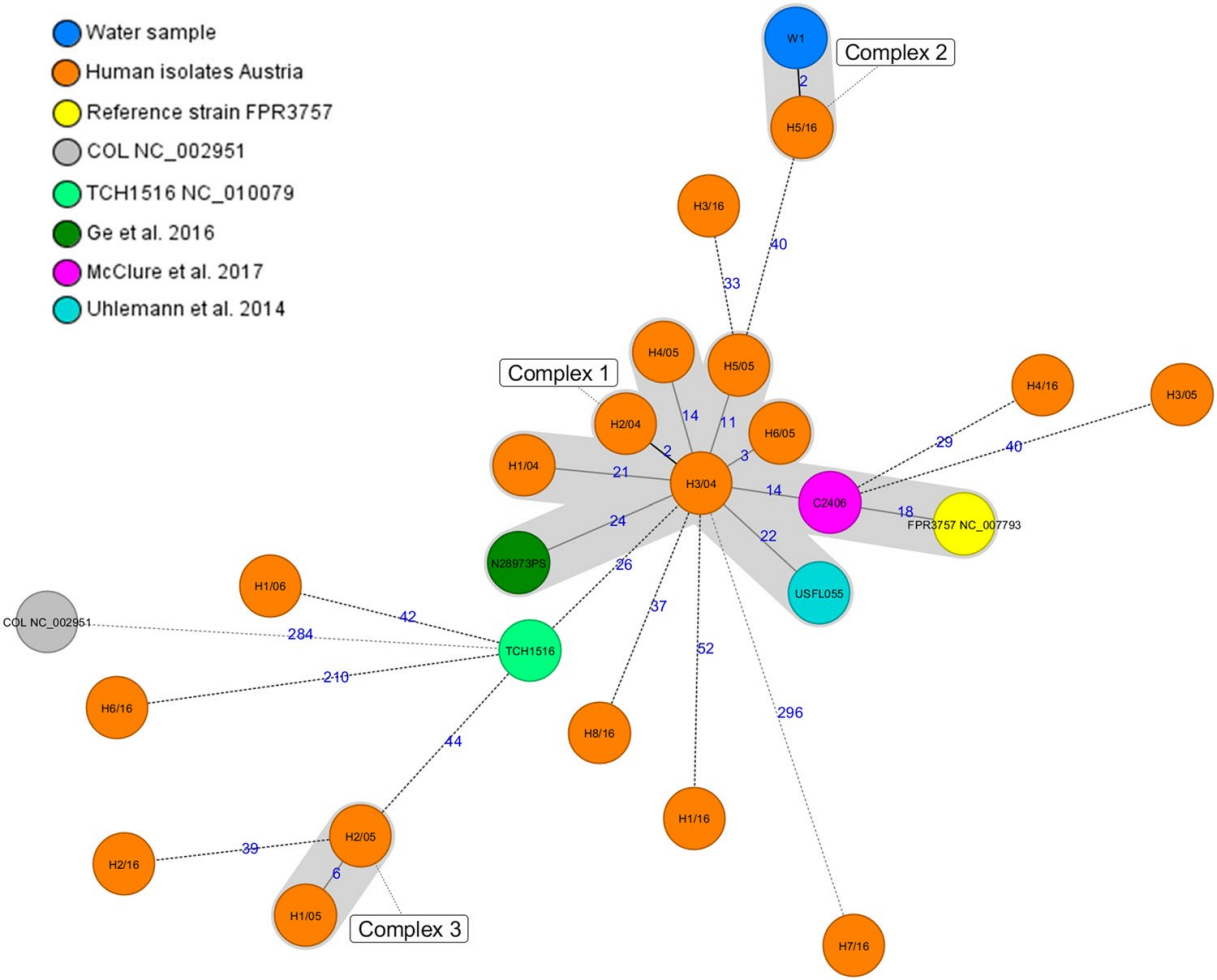
Minimum spanning tree for 25 MRSA isolates based on the cgMLST of *S. aureus*. Colours correspond to the origin of the samples. Each circle represents isolates with an allelic profile based on the sequence of 1,861 core genome targets. Blue numbers refer to the allelic differences between two isolates. Isolates with closely related genotypes were identified with a maximum of 24 allelic differences and are shaded in grey. Austrian isolates: W1, H1/04-H3/04, H1/05-H6/05, H1/06, H1/16-H8/16; reference strains: FPR3757, COL (NC_002951.2); closest related international isolates = C2406^26^, N28973PS^39^, TCH1516^38^, USLF055^36^.

Whole genome based cgMLST phylogenetic analysis including *S. aureus* reference strain COL (accession no. NC_002951.2) (ST250), water isolate (W1), the reference strains FPR3757 (ATCC^®^ BAA-1556^™^), TCH1516 (USA300-HOU-MR, ATCC^®^ BAA-1717^™^), all clinical USA300 isolates (n = 18) from the Austrian national reference laboratory for coagulase positive staphylococci (from 2004, 2005 and 2016), and 143 genomes from isolates retrieved from SRA was performed and a minimum spanning tree was calculated (Fig. 1). Distance calculation between all 164 samples revealed a maximum allelic distance between samples of 351 and an average allelic distance of 93.5 across the MST. Clinical Austrian isolates differed among each other in minimum of two, a maximum of 327, and an average distance of 100 alleles. Based on the defined complex threshold (CT) of 24 allelic differences^18^ 25 different complexes were obtained. Six out of 18 clinical Austrian isolates (H1/04-H3/04; H4/05-H6/05) showed close relatedness with a maximum allelic difference of 21 and were all located in complex 2 (Fig. 1) respectively in complex 1 (Fig. 2). Isolate H3/04, a human isolate from Austria in 2004, showed close relatedness to strains C2406 with an allelic difference of 14, USFL055 isolated in the United States in 2009 with an allelic difference of 22, N28973PS isolated in the United States in 2011 from turkey meat with an allelic difference of 24, and reference strain FPR3757 with an allelic difference of 23. Surface water isolate W1 differed by 54 alleles from C2406 and was closest related to the Austrian clinical isolate H5/16 differing by two alleles in cgMLST and by four alleles in the pan genome including the following targets from the reference genome *S. aureus* (SA)COL (accession no. NC_002951.2): SACOL1678 (luciferase, YP_186518.1, G996T), SACOL1708 (type III leader peptidase, YP_186547.1, C218T) in the core genome and the targets (n = 2) SACOL0289 (hypothetical protein, YP_185183.1, G56T, C57T, G84A, A105C) and SACOL0651 (hypothetical protein, YP_185536.1, C524A) in the accessory genome.

A total of 69 genes were identified referring virulence attributes in USA300 samples W1, H1/04-H3/04, H1/05-H6/05, H1/06, H1/16-H8/16, C2406 and FPR3757. Water isolate W1 harboured 61 virulence gene loci. Comparison of all these 21 isolates revealed 26 identical virulence genes and 60 identical virulence alleles between W1 and H5/16, 59 identical virulence alleles between W1, C2046 and reference strain FPR3757, and 64 identical virulence alleles between H3/04 and C2406 (Supplementary Table S1). All 21 isolates (W1, H1/04-H3/04, H1/05-H6/05, H1/06, H1/16-H8/16, C2406, FPR3757) carried the *agr* allele 1, all except H6/16 and H7/16 carried Panton-Valentine leukocidin (*lukS*-PV/*lukF*-PV), seventeen isolates carried the USA300 characteristic arginine catabolic mobile element (ACME) (W1, H1/04-H3/04, H3/05-H6/05, H1/06, H1/16, H3/16-H6/16, H8/16, C2406, FPR3757), and sixteen isolates carried *seq* (H1/04-H3/04, H2/05-H6/05, H1/06,H2/16-H5/16, H8/16, C2406, FPR3757).

Phenotypic susceptibility testing revealed that the water isolate W1 was resistant to beta-lactams (benzylpenicillin, amoxicillin-clavulanic acid, cefoxitin), fluoroquinolones (ciprofloxacin, moxifloxacin) and erythromycin among the seventeen antibiotics tested (Table 1).

**Table 1:** Phenotypical resistance data for sample W1. MIC (minimum inhibitory concentration) breakpoints accord to EUCAST.

Antibiotic	MIC breakpoint (mg/L)		USA300 W1 (MIC)
	S≤	R>	
Benzylpenicillin	0.125	0.125	R (>32)
Amoxicillin-clavulanic acid	n.a	n.a	R (48)
Cefoxitin	4	4	R (>128)
Ciprofloxacin	1	1	R (>32)
Moxifloxacin	0.25	0.25	R (1.5)
Amikacin	8	16	S (1.5)
Gentamicin	1	1	S (0.125)
Teicoplanin	2	2	S (0.75)
Vancomycin	2	2	S (1)
Erythromycin	1	2	R (32)
Clindamycin	0.25	0.5	S (0.125)
Minocycline	0.5	1	S (0.064)
Linezolid	4	4	S (1.5)
Fosfomycin	32	32	S (0.5)
Fusidic acid	1	1	S (0.125)
Rifampicin	0.06	0.5	S (0.008)
Trimethoprim	2	4	S (0.38)

S = sensitive, R = resistant, I = intermediate, n.a. = not applicable.

In total 25 genes conferring resistance to various antibiotics were identified in USA300 samples W1, H1/04-H3/04, H1/05-H6/05, H1/06, H1/16-H8/16 C2406 and FPR3757. The water isolate W1 harboured in total 14 resistance genes (Table 2) and showed complete concordance to H5/16, sharing nine identical resistance targets with FPR3757 and *fosB* with C2406. SNPs conferring resistance to ciprofloxacin were identified in 15 out of 21 isolates (except H1/04, H1/05, H2/05, H1/06, H2/16, H6/16), due to a L84S change in *gyrA*.

**Table 2:** Identified resistance genes and allele types in USA300 samples W1, H1/04–H3/04, H1/05–H6/05, H1/06, H1/16–H8/16, C2406 and FPR3757.

Target	W1	H1/04	H2/04	H3/04	H1/05	H2/05	H3/05	H4/05	H5/05	H6/05	H1/06	H1/16	H2/16	H3/16	H4/16	H5/16	H6/16	H7/16	H8/16	C2406	FPR3757	Resistance against	Accession Nb.
aadD	ND	ND	ND	ND	ND	ND	ND	ND	ND	ND	ND	ND	ND	ND	ND	ND	ND	1	ND	ND	ND	aminoglycosides	BA000017.4
aphA3	ND	1	1	1	1	1	1	1	1	ND	1	ND	ND	ND	1	ND	ND	ND	1	ND	ND	aminoglycosides	CP009681.1
blaI	7	7	7	7	7	7	7	7	7	7	7	7	7	7	7	7	ND	1	7	ND	ND	beta-lactam	SRR016154
blaR	15	15	15	15	15	15	15	15	15	15	15	15	15	15	15	15	ND	ND	15	6	ND	beta-lactam	SRR016154
blaZ	10	10	10	10	10	10	10	10	10	10	10	10	10	10	10	10	ND	1	10	6	ND	beta-lactam	SRR016154
ccrA2	6	NAT	6	6	6	6	6	6	6	NAT	6	6	6	6	6	6	ND	17	6	1	6	methicillin	NC010079
ccrB2	6	NAT	6	6	6	6	6	6	6	ND	6	6	15	6	6	6	ND	1	6	1	6	methicillin	NC010079
ccrC	ND	ND	ND	ND	ND	ND	ND	ND	ND	ND	ND	ND	ND	ND	ND	ND	NAT	ND	ND	3	ND	methicillin	AP008934.1
dfrA	ND	ND	ND	ND	ND	ND	ND	ND	ND	ND	ND	ND	ND	ND	ND	ND	ND	1	ND	1	ND	trimethoprim	AE017171.1
ermA	ND	ND	ND	ND	ND	ND	ND	ND	ND	ND	ND	ND	ND	ND	ND	ND	ND	1	ND	ND	ND	MLSBK	BA000017.4
fosB	1	1	1	1	1	1	1	1	1	1	1	1	1	1	1	1	1	1	1	1	1	fosfomycin	CP000046.1
lmrP	1	1	1	1	1	1	1	1	1	1	1	1	1	1	1	1	1	1	1	ND	1	various	CP000046.1
mecA	3	3	3	3	3	3	3	3	3	3	3	3	3	3	3	3	4	3	3	1	3	methicillin	BX571856.1
mecR-intact	ND	ND	ND	ND	ND	ND	ND	ND	ND	ND	ND	ND	ND	ND	ND	ND	ND	1	ND	1	ND	methicillin	BA000017.4
mecR-truncated	1	1	1	1	1	1	1	1	1	1	1	1	1	1	1	1	ND	FQC	1	ND	1	methicillin	CP000046.1
merA	ND	ND	ND	ND	ND	ND	ND	ND	ND	ND	ND	ND	ND	ND	ND	ND	ND	1	ND	ND	ND	SCCmec	NC013352.1
merB	ND	ND	ND	ND	ND	ND	ND	ND	ND	ND	ND	ND	ND	ND	ND	ND	ND	1	ND	ND	ND	SCCmec	AB179623.1
mphC	2	2	2	2	2	2	2	2	2	2	2	2	FQC	FQC	2	2	ND	ND	2	ND	ND	MLSBK	NC017351
mprF	1	1	1	1	1	1	1	1	1	1	1	NAT	1	1	1	1	1	1	1	ND	1	methicillin-oxacillin	CP000046.1
msrA	2	2	2	2	2	2	2	2	2	2	2	2	2	FQC	2	2	ND	ND	NAT	ND	ND	MLSBK	NC017351
qacA	ND	ND	ND	ND	ND	ND	ND	ND	ND	ND	ND	ND	ND	ND	ND	ND	ND	NAT	ND	ND	ND	various	AF053771.1
sdrM	1	1	1	1	1	1	1	1	1	1	1	1	1	1	1	1	1	1	NAT	ND	1	various	CP000046.1
tetK	ND	ND	ND	ND	ND	ND	ND	4	ND	ND	ND	ND	ND	ND	ND	ND	ND	ND	ND	ND	ND	tetracycline	sampleLGO017
ugpQ	1	1	1	1	1	1	1	1	1	1	1	1	1	1	1	1	1	1	1	ND	1	SCCmec	CP000046.1
xylR	ND	ND	ND	ND	ND	ND	ND	ND	ND	ND	ND	ND	ND	ND	ND	ND	ND	2	ND	ND	ND	SCCmec	BA000018.3

Numbers accord to the respective allele type for each target; FQC = failed target QC procedure; NAT = new allele type; ND = not detected.

The presence of antimicrobial resistance against penicillin, methicillin, erythromycin and ciprofloxacin in isolate W1 was confirmed by in silico analysis using Mykrobe predictor.

Comparative analysis of isolate W1 to the closest relative isolate H5/16 via OrthoFinder revealed following differences in presence and absence of protein coding genes: additional presence of two domain-containing proteins (DUF443) and one hypothetical protein in isolate W1 and the additional presence of ten hypothetical proteins, four transposases, two family proteins (TIGR01741), one domain-containing protein (DUF5079), one immunoglobulin G-binding protein A and one transcriptional regulator in isolate H5/16.

Comparison of isolate W1 to isolate C2406 revealed nine additional hypothetical proteins, three domain-containing proteins (DUF443 (n = 2), DUF536), three transcriptional regulators, three transferases, two transporter, one penicillinase repressor BlaI, beta-lactam sensor/signal transducer BlaR1, penicillin-hydrolyzing class A beta-lactamase BlaZ, quinone oxidoreductase, one transposase and one ABC-F type ribosomal protection protein MsrA in isolate W1 and additional eight hypothetical proteins, two family proteins (TIGR01741), two transposases, one domain-containing protein (DUF5079) and one transcriptional regulator in isolate C2406 (Supplementary Table S2).

Additionally 15 genes in isolate W1 were identified for which no orthologue could be assigned in the other genomes in this comparison: two ATP-binding proteins, two family proteins (TIGR01741), two hypothetical proteins, one acyltransferase, one LysR family transcriptional regulator, one AI-2E family transporter, one homoserine dehydrogenase, one recombination protein RecJ and one DUF5079 domain-containing protein. Three genes revealed no significant result and could not be assigned to any known group of protein coding genes (Supplementary Table S3).

**Figure 3:**
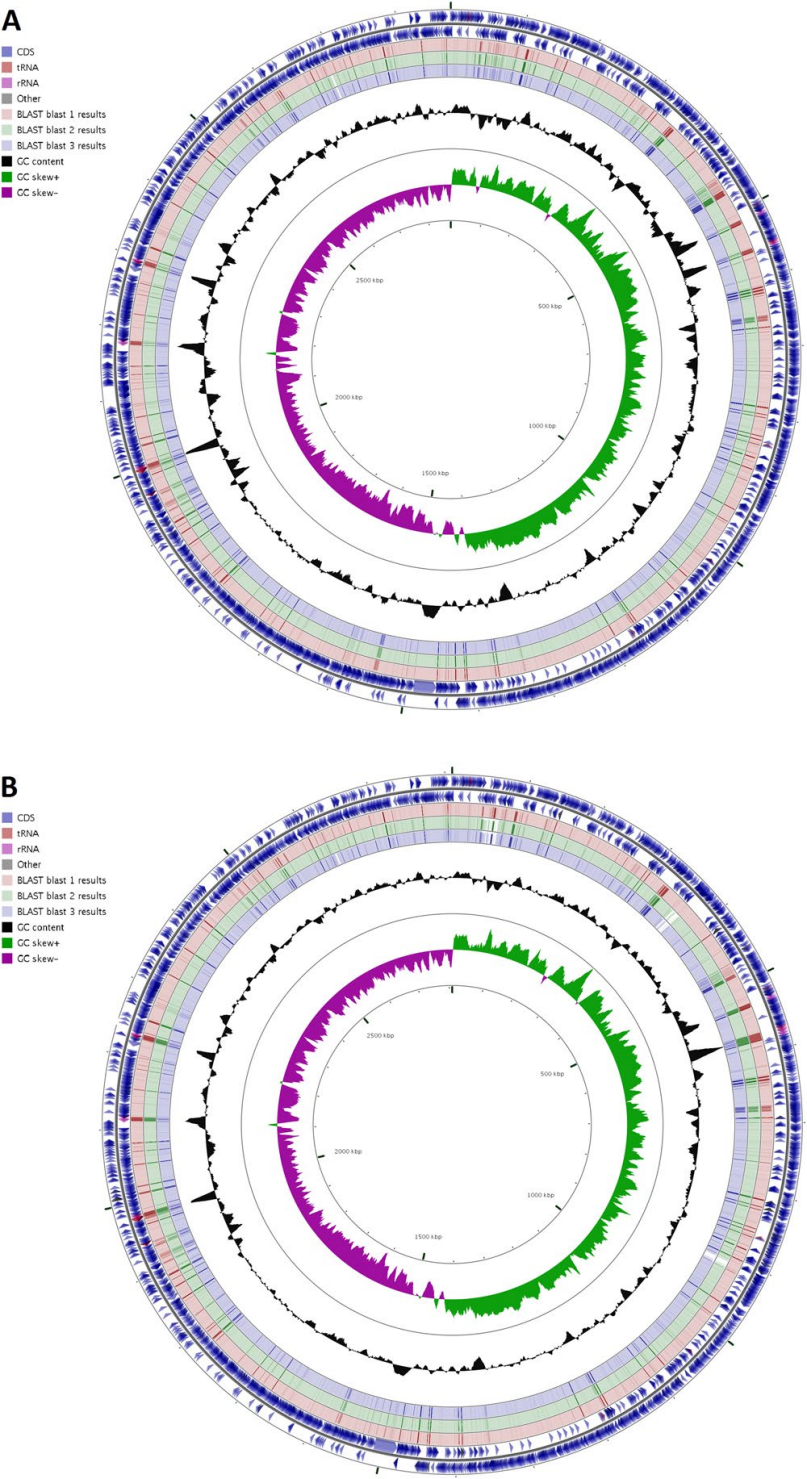
Comparative circular maps of *Staphylococcus aureus* genomes USA300_FPR3757 (NC_007793) and COL (NC_003951.2) to USA300 isolates. (**A**) The circles display the following information (from outside to inside): CDSs of USA300_FPR3757 on the + strand, CDSs of USA300_FPR3757 on the − strand, blastn result 1: USA300_FPR3757, blastn result 2: H5/16, blastn result 3: W1, GC content, GC skew + (green) and − (violet) value; (**B**) The circles display the following information (from outside to inside): CDSs of COL (NC002951.2) on the + strand, CDSs of COL (NC002951.2) on the − strand, blastn result 1: COL (NC002951.2), blastn result 2: USA300_FPR3757, blastn result 3: W1, GC content, GC skew + (green) and − (violet) value.

The circular map (CGView Server V 1.0 (2007)) depicts sequence similarity between the reference strain FPR3757, isolates H5/16 and W1 and sequence similarity between COL (NC_002951.2), FPR3757 and isolate W1 in Fig. 3.

## Discussion

The discharge, persistence and dissemination of AMR in nature are considered a major public health threat worldwide^19^. Therefore a strategy to better control the release of antibiotics into the environment and subsequently to prevent contact of bacteria from human and animal sources with environmental organisms^20^ is of utmost importance.

*Staphylococcus aureus* was particularly successful in developing resistance to antibiotics. MRSA is listed as of high priority on WHOs antibiotic-resistant pathogens list (http://www.who.int/medicines/publications/global-priority-list-antibiotic-resistant-bacteria/en/). Since the first detection of MRSA with association to healthcare (HA-MRSA) settings in 1961^21^, new epidemic MRSA clones have emerged affecting the community (CA-MRSA) or being associated with animals (LA-MRSA)^22–24^. Surveillance and characterisation of clinical, animal and environmental isolates is a public health imperative.

The MRSA isolate described was obtained from a water sample from a small river in southern Austria^24^. Characterisation of this isolate by WGS analysis revealed that the isolate has all characteristic features of CA-MRSA USA300^7^. CgMLST and wgMLST based gene-by-gene comparison^18^ to eighteen clinical isolates (USA300) available from the National Reference Laboratory for Staphylococci including *Staphylococcus aureus* revealed a very close relatedness to one clinical USA300 isolate (H5/16) collected 2016 in Vienna, differing only by two alleles in their core- and by four alleles in their whole-genome targets. The water and patient isolates shared 60 identical virulence and 14 antibiotic resistance alleles. We were unable to find an epidemiological link between these two isolates. Four isolates collected in 2016 from a hospital located in one of the settlements next to this river, showed no close relatedness to the USA300 water isolate.

An interesting additional outcome of this WGS based typing study was that clinical USA300 isolates from Austria are a diverse population indicating that USA300 clones were introduced several times in Austria and are not all descendants from the first Austrian USA300 isolate from 2004. According to the WGS patterns all eight clinical Austrian USA300 isolates from 2016 are unrelated to each other. Thus in comparison to previous typing methods that did not allow a differentiation (same MLST, same *spa* type), WGS based typing is an important improvement representing an added information value for hospitals allowing a clear discrimination of outbreak and transmission events from unrelated isolates. In due consideration of staphylococcal mutation rates of one SNP per 15 weeks^25^ we can assume that the water isolate W1 and several clinical isolates from Austria are descendants of the complex containing the hypervirulent USA300-C2406 strain from a patient with necrotizing pneumoniae isolated in Canada in 2004^26^ and reference strain FPR3757^1^. In contrast to the aforementioned Austrian USA300 isolates from 2016, the first USA300 isolates detected in Austria in 2004 to 2005^10^ belong to four different complex types. One complex contained isolates collected from two patients’, stationary in the same hospital in 2005, whereof the initial one was derived from a postal worker processing post from the United States, which had a clear transmission link. The second complex contained six isolates indicating a transmission or a common source of infection. Within this complex an epidemiological link to the United States could only be confirmed for one isolate (isolate ID H4/05) as well as the close relatedness between a couple (isolate ID H2/04 & H3/04) and an Austrian isolate from 2005 (isolate H6/05). CgMLST analysis shows that Austrian isolates of the second complex are closely related to several US isolates including C2046 and reference strain FPR3757 isolated from a HIV-positive patient in the United States in 2000^1^ demonstrating a transfer. In contrast to the United States, where USA300 has become the most successful MRSA clone in the community as well as in hospitals, USA300 is still rare in most European countries and Austria. However, the fact that USA300 infections transmitted via contaminated environmental sources have been demonstrated^27^ and the detection in a river water sample with close relatedness of the water isolate to a clinical isolate might indicate a possible risk for public health.

In conclusion, this is to our best knowledge the first genome based characterisation of an environmental USA300 isolate confirming the emerging global health threat caused by environmental AMR. While numerous environmental AMR studies have reported on Gram negative bacteria our example shows that also Gram positive bacteria might play their role. Considering the historical evolution of MRSA clones the emergence of new pathogenic clones is merely a question of time.

For appropriate actions further investigations are essential to determine the occurrence, risk, environmental impact of AMR in the environment as well as use of optimal wastewater treatment technologies to alleviate this public health threat^28^.

## Methods

### Microorganisms and Species Identification

In 2016 we collected 12 water samples from four rivers (Danube (n = 3, 48°14′53″N, 14°25′ 37″E; 48°14′45″N, 14°26′1″E; 48°14′32″N, 14°26′41″E), Glan (n = 3, 46°42′46″N, 14°6′23″E; 46°38′1″N, 14°18′56″E; 46°38′14″N, 14°17′44″E), Inn (n = 4, 47°16′35″N, 11°12′50″E; 47°15′55″N, 11°16′58″E; 47°16′14″N, 11°26′16″E; 47°16′6″N, 11°27′42″E), Traun (n = 1, 48°13′2″N, 14°15′26″E)) flowing through the Austrian provinces Carinthia, Salzburg, Tyrol, Lower Austria, Upper Austria, and Vienna. Samples were collected in sterile 500 ml sodium thiosulfate containing bottles (Corning^®^ Gosselin™, NY, USA), 20 to 30 cm below the river surface and 50 to 100 cm apart from the river bank. For MRSA screening 100 ml aliquots of samples were filtrated and bacterial isolates were enriched by incubating the filter in tryptone soya broth (containing 3.5 mg/ml cefoxitin and 75 mg/ml aztreonam) at 37 °C overnight according to the standard protocol for isolating MRSA from dust samples (http://www.eurl-ar.eu/233-protocols.html, Isolation of MRSA from dust samples). To minimize the risk of contamination water sample filtration and cultivation were separated spatially, locally and temporally from cultivation of clinical samples. Laboratories were weekly screened for contaminations using air settlement plates and surface swabbing according to a standard operation protocol (SOP) following international guidelines^29^. For detection of methicillin resistant staphylococci, the overnight cultures were cultivated on BBL™ CHROMagar™ MRSA II according to the manufacturer’s instructions (Becton Dickinson, Vienna, Austria). Mauve colonies from chromogenic media were sub-cultured and identified by matrix assisted laser desorption/ionisation time-of-flight (MALDI-TOF) mass spectrometry.

The clinical MRSA USA300 isolates from the Austrian National Reference Laboratory for Coagulase Positive Staphylococci originating from 2016 (H1/16 to H4/16) came from a hospital located in a settlement beside the river (1,470 beds) in Carinthia and four isolates (H5/16 to H8/16) were from Vienna and from the province Lower Austria. Additionally isolates from the Austrian provinces Vienna, Lower Austria, Salzburg and Styria from 2004 (H1/04-H3/04), 2005 (H1/05-H6/05) and 2006 (H1/06) were analysed.

### Antibiotic susceptibility testing

*In vitro* susceptibility was tested for 17 antibiotics (benzylpenicillin, amoxicillin-clavulanic acid, cefoxitin, ciprofloxacin, moxifloxacin, amikacin, gentamicin, teicoplanin, vancomycin, erythromycin, clindamycin, minocycline, linezolid, fosfomycin, fusidic acid, rifampicin, trimethoprim) by Etests (bioMérieux, Marcy-l'Ètoile, France) and interpreted according to EUCAST (European Committee on Antimicrobial Susceptibility Testing, EUCAST Clinical Breakpoint Tables v.7.1, valid from 2017-03-10).

### Whole genome sequencing and data analysis

For whole genome sequencing (WGS) high quality genomic DNA (gDNA) was isolated using the MagAttract HMW DNA Kit (Qiagen, Hilden, Germany) and quantified with a Qubit^®^ 2.0 Fluorometer (Thermo Fisher Scientific, Waltham, MA, USA) using the dsDNA BR Assay Kit (Thermo Fisher Scientific, Waltham, MA, USA). Libraries of bacterial genomes were prepared using Nextera XT DNA Library Preparation Kit (Illumina, San Diego, CA, USA) according to the manufacturer’s protocol. A desired coverage of at least 50-fold was calculated with Sequencing Coverage Calculator (www.illumina.com/CoverageCalculator) and bacterial isolates were paired-end sequenced with a read length of 2 × 300 basepairs on a MiSeq instrument (Illumina, San Diego, CA, USA). SPAdes version 3.9.0^30^ was used for read assembly and SeqSphere^+^ version 4.1.9 (Ridom, Münster, Germany) for strain characterisation using a recently published cgMLST scheme^18^ and calculation of minimum spanning trees (MST). Phylogenetic relatedness between water and related clinical isolates was further visualized using CGView Server V 1.0(2007)^31^.

*Spa* type^32^, MLST (multilocus sequence type)^33^, cgMLST (core genome MLST)^18^, resistance genes and virulence genes^34^ were extracted from WGS data using SeqSphere^+^ version 4.1.9. Mutations in *cap5* were identified according to the publication of Boyle-Vavra *et al*. (2015) and mutations in *gyrA* causing ciprofloxacin resistance were identified according to Sreedharan *et al*.^35,36^. Genotype-based antimicrobial-resistance was predicted for isolate W1 by using Mykrobe predictor for *Staphylococcus aureus*^37^.

### Genomes from Sequence Read Archive

One-hundred and forty-six isolates downloaded from the Sequence Read Archive (SRA) belonged to following Bioprojects: PRJEB2870 (n = 28)^38^, PRJEB3174 (n = 55)^11^, PRJNA224116 (n = 3)^1,39,40^, PRJNA239000 (n = 6), PRJNA268482 (n = 1), PRJNA268547 (n = 1), PRJNA275322 (n = 5), PRJNA311554 (n = 43)^41^, PRJNA341781 (n = 3), PRJNA345240 (n = 1)^26^.

### Analysis of gene presence and absence

Detailed analysis of additional genomic information was performed for 10 genomes: Reference strains C2406 (CP019590.1) and FPR3757 (CP000255.1) and isolates H1/04, H2/04, H3/04, H4/04, H5/04, H6/04, H05/16 and W1. The contigs of each assembly was filtered for a minimum length of 1,000 nucleotides. Genes were predicted using prodigal v. 2.6.3^42^ with default parameters, orthologous groups where calculated with OrthoFinder v. 1.1.4^43^. Orthologous groups with differences in presence/absence were selected. Annotation of the orthologous groups and genes without group assignment was performed using NCBI-BLASTp v. 2.6.0+ with e-vaule cutoff of 0.01^44^ and the RefSeq nr-protein database release 84^45^. The most frequent annotation in an orthologous groups was selected as description for the group.

### Nucleotide accession number(s)

This Whole Genome Shotgun project including ST8 isolates (H1/16-H8/16) from the Austrian national reference laboratory for coagulase positive staphylococci in 2016 and the water isolate (W1) have been deposited at DDBJ/ENA/GenBank under the accession NKCP00000000 to NKCX00000000. The version described in this paper is version NKCP01000000 to NKCX01000000.

### Data availability

All data generated or analysed during this study are included in this published article.

## Electronic supplementary material

Supplementary Table S1

Supplementary Table S2

Supplementary Table S3
